# Deubiquitinase Inhibitors Impair Leukemic Cell Migration Through Cofilin Oxidation and Alteration of Actin Reorganization

**DOI:** 10.3389/fphar.2021.778216

**Published:** 2022-01-07

**Authors:** Frédéric Larbret, Pierric Biber, Nicholas Dubois, Stoyan Ivanov, Laurence Lafanechere, Sophie Tartare-Deckert, Marcel Deckert

**Affiliations:** ^1^ Université Côte d’Azur, INSERM, C3M, Nice, France; ^2^ Team MicroCan, Equipe labellisée Ligue Contre le Cancer, Nice, France; ^3^ Université Grenoble Alpes, INSERM, Institut pour l'Avancée des Biosciences, La Tronche, France

**Keywords:** actin cytoskeleton, FRET, DUB, cofilin, leukemic cell migration

## Abstract

Actin networks are dynamically regulated through constant depolymerization and polymerization cycles. Although the fundamental mechanisms that govern these processes have been identified, the nature and role of post-translational modifications (PTMs) of actin and actin regulatory proteins are not completely understood. Here, we employed Actin CytoFRET, a method that we developed for real time detection of fluorescence resonance energy transfer (FRET) signals generated by actin dynamics, to screen a small library of PTM-interfering compounds on a biosensor leukemic T cell line. This strategy led to the identification of small molecule inhibitors of deubiquitinating enzymes (DUBs) as potent inducers of actin polymerization and blockers of chemotactic cell migration. The examination of the underlying mechanism further revealed that the actin depolymerizing protein cofilin represents a major effector of DUB inhibitor (DUBi)-induced actin reorganization. We found that DUB blockade results in the accumulation of polyubiquitinated proteins and ROS production, associated with cofilin oxidation and dephosphorylation on serine 3, which provokes uncontrolled actin polymerization impairing cell migration. Together, our study highlights DUBs as novel regulators of actin dynamics through ROS-dependent cofilin modulation, and shows that DUBi represent attractive novel tools to impede leukemic cell migration.

## Introduction

Dynamic remodeling of actin structures allow cells to respond to extracellular stimuli and mechanical cues and occur during major cell biological processes, including cytokinesis, endocytosis, adhesion and migration (Pollard and Cooper, 2009; Tojkander et al., 2012). Consequently, actin cytoskeleton dysfunction has been implicated in many pathological conditions including immunological diseases and cancer. Actin, one of the most abundant proteins found in all eukaryotic cells, exists under two different forms: a monomeric globular form (G-actin) and a polymeric filamentous form (F-actin). Spontaneous actin polymerization follows the hydrolysis of a bound adenosine triphosphate (ATP) molecule. However, an equilibrium between G-actin and F-actin is maintained through continuous actin filaments treadmilling, in which monomers dissociate from the pointed end and polymerize at the barbed end (Suarez and Kovar, 2016). The shaping of complex and dynamic actin filament networks is controlled by actin regulatory proteins displaying specialized functions such as actin monomers sequestration, filament nucleation, elongation and branching, as well as filament capping, severing and crosslinking (Paavilainen et al., 2004; Rotty et al., 2013; Pollard, 2016). In addition, small GTPases of the Rho superfamily provides a link between extracellular environment and the actin machinery (Sit and Manser, 2011).

Post-translational modifications (PTMs) of actin emerge as major regulatory events of actin dynamics (Terman and Kashina, 2013). Specific covalent modifications by a vast array of enzymes include acetylation, methylation, ADP-ribosylation, arginylation, phosphorylation and ubiquitination. These PTMs, as well as other redox-related modifications such as oxidation, differentially alter actin cytoskeleton remodeling in both physiological and pathological situations (Schaefer et al., 2012; Terman and Kashina, 2013). Adding to the complexity, actin regulatory proteins are also subjected to PTM. For example, tyrosine phosphorylation of WASp is linked to signal-enhanced actin polymerization (Cory et al., 2003; Badour et al., 2004). Actin binding of the actin-depolymerizing factor (ADF)/cofilin family is modulated by PTMs, including phosphorylation on serine 3 (Bernstein and Bamburg, 2010; Kanellos and Frame, 2016) and oxidation (Klemke et al., 2008; Cameron et al., 2015). Recent studies have shown the importance of ubiquitination/deubiquitination of ABPs such as WASH and Coronin 7 for the regulation of actin assembly and protein trafficking (Hao et al., 2013; Yuan et al., 2014). Also, the activity and expression of RhoA are regulated through phosphorylation and ubiquitination (Lang et al., 1996; Wang et al., 2003). However, the role and interplay between the multiple PTMs of actin and actin regulatory proteins are still largely misunderstood.

In this study, we examine the effect of pharmacological modulators of PTMs on actin remodeling by using Actin CytoFRET, a method that we developed for the detection of FRET signals generated by actin polymerization/depolymerization (Larbret et al., 2013). The major advantage of this approach is that it makes it possible to measure not only the degree of polymerized actin (F-actin), comparable to phalloidin labeling, but also the actin nucleation allowing to better evaluate the steady-state dynamics of actin filaments. The screening of a small library of PTM-interfering chemical compounds on the Actin CytoFRET leukemic biosensor T cell line Jurkat identified multiple inhibitors of deubiquitinating enzymes (DUBs) as potent inducers of actin reorganization. DUBs represent a large group of ubiquitin-specific proteases that are involved in essential processes, such as ubiquitin recycling, protein trafficking and activity and protein degradation by the ubiquitin-proteasome system (UPS) (Komander et al., 2009; D'Arcy et al., 2015). Several DUB inhibitors (DUBi) were found to trigger a rapid and dose-dependent actin reorganization, and to impair leukemic cell chemotactic migration. Mechanistically, we found that actin reorganization triggered by DUB blockade involved the dephosphorylation and oxidation of the actin remodeling protein cofilin. These findings provide evidence that DUB inhibitors induce a strong oxidative burst impacting actin cytoskeleton reorganization and cell migration, thereby suggesting their use as potential anti-leukemic therapies.

## Materials and Methods

### Cells, Culture, Antibodies and Reagents

The Jurkat leukemic T cell line (clone E6.1) was from American Type Culture Collection (ATCC, catalog number TIB-152). Jurkat cells were cultured at 37°C in 5% CO_2_ in RPMI supplemented with 10% fetal bovine serum (FBS) (Hyclone), and DMEM, 10% fetal bovine serum, respectively). Control (CT) and Actin CytoFRET (ACT) reporter cell lines were generated as previously described (Larbret et al., 2013). Antibodies against β-actin (C4) were purchased from Santa Cruz Biotechnology, cofilin (D3F9), ERK2 (9108), profilin (C56B8), ubiquitin (P4D1), and phospho-ADF/cofilin (3312) were purchased from Cell Signaling Technology. Anti-gelsolin (ab11081) was from Abcam. Anti-thymosin β4 (AF6796) and recombinant human CXCL12/SDF-1 were from R&D Systems. WP1130, b-AP15 and PR-619 were from Merck. VLX1570 and AMD3100 were from Selleckchem. All other chemicals were from Merck. Plasmid DNA were transfected with Lipofectamine 2000 (Thermo Fischer Scientific).

### Plasmid Constructions

Plasmid constructions, cloning, and DNA sequencing were carried out according to standard protocols. For single color (EGFP or mOrange) and EGFP or mOrange fused to actin, cloning strategies were described previously (Larbret et al., 2013).

### Cofilin shRNA Lentiviral Particles

Cofilin-1 shRNA and control shRNA lentiviral particles were purchased from Santa Cruz Biotechnology. To generate cofilin-1 knockdown cells, 1 × 10^5^ Jurkat cells were infected with 10 μl of lentiviral particles encoding either non targeting control shRNA sequence or cofilin shRNA for 12 h, washed and cultured for 48 h. After a second infection cycle, cells were cultured with puromycin to select infected cells. Transduction and shRNA efficiency was assessed by immunoblotting with antibody against cofilin.

### FRET Measurement and Actin CytoFRET Screening Protocol

Actin FRET signals were recorded on actin biosensor cell line (FRET-ACT) using a ratiometric method (Larbret et al., 2013). Briefly, untreated or treated FRET-CT and FRET-ACT cell lines were analyzed on a FACSCanto II flow cytometer (BD Biosciences) by using 488 nm donor excitation wavelength. Donor fluorescence was collected in the EGFP channel with a 530/30 nm filter, while mOrange acceptor signal was measured with a 610/15 nm filter. FRET ratio was determined as the ratio between acceptor (mOrange) and donor (EGFP) fluorescence intensity under the donor excitation wavelength. The FRET ratio that is determined in untreated or DMSO-treated FRET-ACT cells reflects the degree of basal actin polymerization. For each sample, a minimum of 5,000 cells that fell within the adjusted morphology gate was analyzed. For real time flow cytometry analysis, measurements were performed using an ARIA II cytometer (BD Biosciences) equipped with a heating sample injection chamber that maintains samples at 37°C during the analysis.

For treatment with chemical compounds, FRET-CT and FRET-ACT cell lines were seeded in 96 well plates at a density of 1 × 105 cell/ml the day before FACS analysis. Cells were then incubated with each compound or DMSO for 1 h at 37°C before flow cytometry analysis. A FACSCanto II flow cytometer equipped with a carousel loader was used. Each compound was screened at three different concentrations, with DMSO treatment as control. To calculate actin FRET efficiency in the presence of a compound, we used the following formula: Compound FRET efficiency (%) = [1-(DMSO FRET ratio/Compound FRET ratio)] × 100.

### ROS Staining

CM-H_2_DCFDA (Thermo Fisher Scientific) was dissolved in DMSO and stored as 100 mM at –20°C. Cells were incubated with 10 μM CM-H_2_DCFDA for 30 min at 37°C. Then, cells were washed twice with PBS and exposed to pharmacological treatments during 1 h at 37°C. CM-H_2_DCFDA fluorescence was detected by flow cytometry with an excitation wavelength of 488 nm and emission wavelength of 535 nm.

### Apoptosis Analysis

Cell death was evaluated using an Apoptosis Detection Kit (BD Biosciences) as described previously (Didier et al., 2018). Briefly, 10^5^ cells were washed twice with PBS, stained with Annexin-V and propidium iodide in binding buffer for 15 min at room temperature. Cells were then analyzed by flow cytometry using a FACSCanto II and the DIVA8 software (BD Biosciences).

### Aggresome Staining

Proteostat aggresome detection kit (Enzo Life Sciences) was used for aggresome staining. Briefly, Jurkat cells were washed with Ice cold PBS, then fixed with 3.7% formaldehyde in PBS, and permeabilized with 0.5% Triton in PBS, 1% bovine serum albumin for 20 min at room temperature. Cells were washed and labeled with 200 μl of proteostat red dye and incubated for 30 min at room temperature. Analysis was performed by flow cytometry.

### Cell Lysis and Immunoblotting

Cells were lysed in ice-cold RIPA lysis buffer: 1% NP-40 in 50 mM NaCl, 50 mM Tris-HCl, pH 7.4, 0.1% SDS, 0.5% sodium deoxycholate, 150 mM NaCl, 2 mM EDTA, 50 mM NaF supplemented with protease and phosphatase inhibitors (Roche Diagnostics) for 30 min on ice. Then, samples were centrifuged at 7,500 g for 10 min at 4°C. Supernatant (soluble fraction) was separate from pellet (insoluble fraction). Oxyblot detection kit (Abcam) was used for the detection of cofilin oxidation. The procedure for derivatization of protein carbonyls with DNPH and subsequent detection using the Oxyblot kit basically follow the procedure as outlined in the kit brochures. Briefly, 20 μg protein was derivatized with DNPH and separated on a 10% pre-cast gel (BioRad, United States) using standard SDS polyacrylamide electrophoresis. Following separation, the gels were transferred to a PVDF membrane and probed with the primary polyclonal anti-DNP and secondary antibodies anti-rabbit IgG provided in the kit and developed using enhanced chemiluminescence (GE Healthcare).

### GFP-Trap Immunoprecipitation

Jurkat T cells were electroporated with GFP-cofilin expression vector (Addgene Plasmid #50859). 48 h post transfection cells were lysed in ice cold RIPA lysis buffer supplemented with 10 mM DTT. 2 ml of clarified lysate was incubated with 50 µl of GFP-Trap^®^ (Chromotek) for 1 h at 4°C, and washed three times in IP buffer for 5 min each. The washed GFP-Trap^®^ (Chromotek) resin was incubated for 2 min in IP buffer adjusted to pH 2.0 in order to acid-elute the samples.

### Cell Migration Assay

Jurkat cell migration was assessed on uncoated polycarbonate Transwell-96 well support plates with 5-μm pore size (Corning). Cells were serum-starved for 4 h before the experiment. 1 h before the experiment, chemical compounds were added to the medium. Then, medium alone or CXCL12 (100 ng/ml) was added in the lower chamber, and the treated cell suspension (5 × 10^5^ cells) was added in the upper chamber. Cell migration in the lower well was measured after 90 min of migration and quantified by flow cytometry as described before (Ticchioni et al., 2002).

### Immunofluorescence and Microscopy Analysis

Jurkat cells were adhered on poly-l-lysine coated slides (Thermo scientific), fixed with 3.7% paraformaldehyde. Cells were then permeabilized with 0.1% Triton X-100, washed and incubated overnigh with PBS supplemented with 1% bovine serum albumin (BSA) in the presence of anti-Cofilin antibodies. Following incubation with Alexa Fluor 594-conjugated goat anti-rabbit cross-adsorbed secondary antibodies (Thermo Fischer Scientific) for 30 min and nuclei staining with DAPI, coverslips were mounted in ProLong antifade mounting reagent (Thermo Fisher Scientific). Slides were then analyzed on a Axiovert 200M microscope (Zeiss) equipped with Orca CCD camera (Hamamatsu).

### Statistical Analysis

All data are presented as the mean ± SD of at least three independent determinations. *p*-values were determined using the Prism software (GraphPad, United States). Data sets were tested for normality, no outliers were removed and Student’s T tests were performed to determine statistical significance. *p*-values of 0.05 (*), 0.01 (**) and 0.001 (***) were considered statistically significant.

## Results

### Actin CytoFRET Screening Identifies DUB Inhibitors as Actin Cytoskeleton Modulators

**FIGURE 1:**
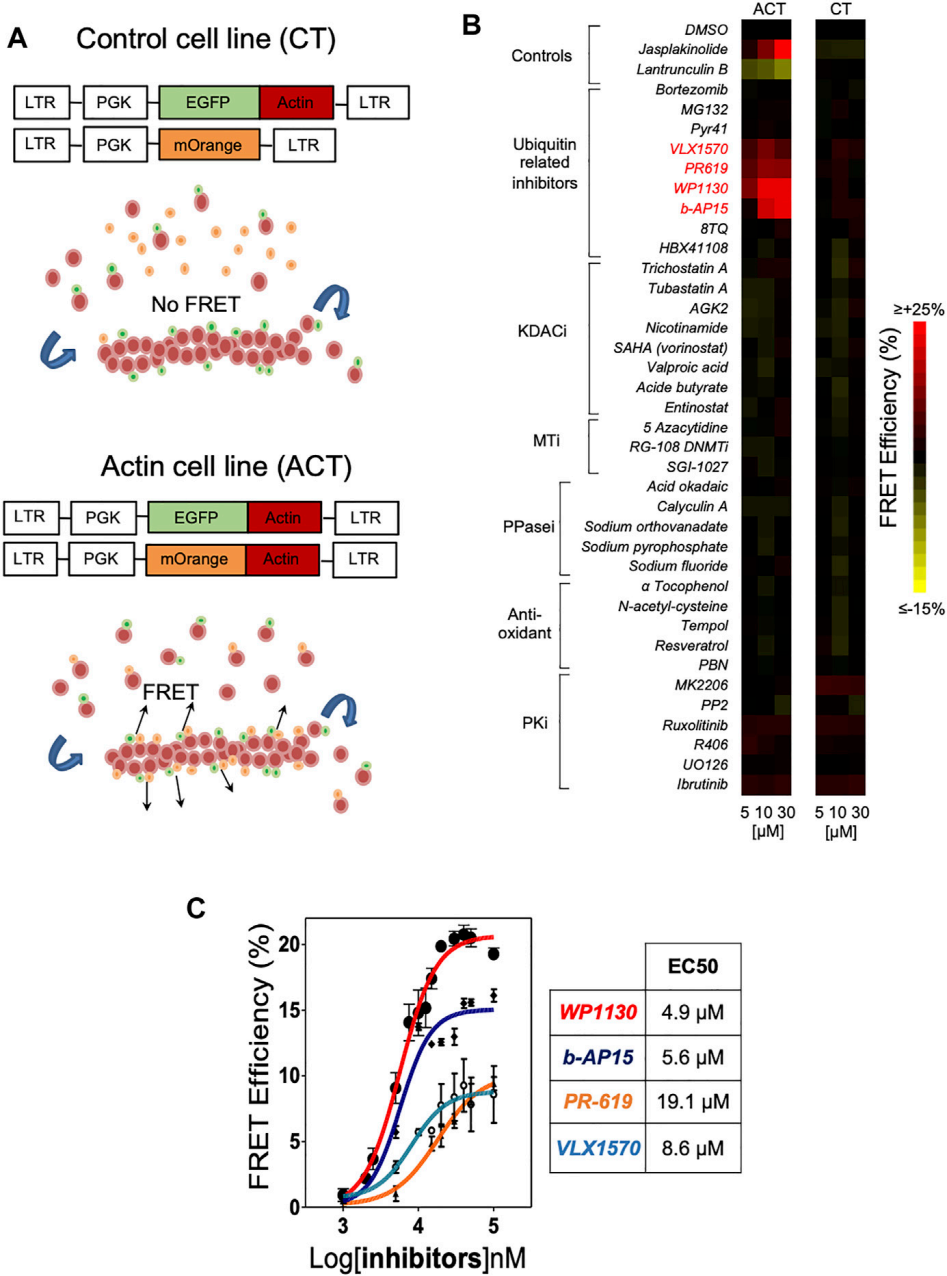
High-content screening using Actin CytoFRET identifies deubiquitinase inhibitors as novel modulators of actin remodeling. **(A)** Actin CytoFRET principle: Actin-FRET (ACT) cell line expresses EGFP-actin and mOrange-actin fluorescent proteins (FPs). FRET signals are emitted in FRET-ACT cells when EGFP-actin and mOrange-actin locate close to each other in actin filaments. FRET-control (CT) cell line expresses EGFP-actin and mOrange alone that cannot be incorporated into actin filaments, preventing FRET signal emission. FRET signals are monitored by flow cytometry using a ratiometric method. **(B)** Heatmap representation of Actin CytoFRET screening of a small library of compounds affecting post-translational modifications. Each compound was tested at three concentrations (5, 10, 30 µM) for 60 min on ACT and CT reporter cell lines and FRET signals were recorded by flow cytometry. Data are expressed as the percentage of FRET efficiency between DMSO-treated and compound-treated cells. Jasplakinolid and Latrunculin B were tested as controls for actin polymerization and depolymerization, respectively. **(C)** Dose-response determination of the activity of DUBi WP1130, b-AP15, PR-619 and VLX1570 on actin FRET efficiency. Data represent the mean ± SD of *n* = 3 independent experiments. EC50 for each drug were calculated using GraphPad Prism software.

In order to evaluate the impact of post-translational modifications on actin dynamics, we employed Actin CytoFRET, a method that we developed for real-time detection by flow cytometry of FRET signals generated by actin filament remodelling in engineered Jurkat leukemic T cell lines (Larbret et al., 2013). Briefly, the principle of the method is based on the use of biosensor cell lines expressing actin monomers fused to EGFP or mOrange fluorescent proteins. A cell line (CT) expressing actin-EGFP and unfused mOrange serves as a negative control (Figure 1A). Within the Actin CytoFRET cell line (ACT), the interaction of actin-EGFP and actin-mOrange during actin assembly generates a FRET signal that is recorded by flow cytometry using a ratiometric method (Larbret et al., 2013). Increase of FRET signal is characterized by a decrease in GFP fluorescence intensity and an increase in mOrange intensity upon 488 nm wavelength excitation laser. The actin stabilizing agent Jasplakinolide and the actin depolymerizing drug Latrunculin B were used as controls for monitoring actin assembly and disassembly, respectively. While Jasplakinolide induced an increase of FRET signals, Latrunculin B resulted in a decrease of FRET signals (Figure 1B). Using this method, we screened a small library of compounds interfering with PTMs such as acetylation, methylation, oxidation, phosphorylation and ubiquitination for their activity on actin cytoskeleton. ACT and CT cell lines were incubated with either DMSO or increasing doses of the indicated compounds. The majority of tested molecules had no effect on actin dynamics. On the contrary, the treatment with four inhibitors of DUBs, WP1130, b-AP15, VLX1570 and PR-619 (Supplementary Figure S1) induced a strong dose dependent increase of actin FRET signals that compared to the effect of Jasplakinolide (Figure 1B). At a concentration of 30 μM, FRET signals were augmented by WP1130, b-AP15, VLX1570 and PR-619 by 20.3, 15.8, 7.6 and 8.1%, respectively (Figure 1B). Dose responses performed with these compounds showed that the EC50 for increasing actin FRET efficiency was 4.9, 5.6, 8.6 and 19.1 μM for WP1130, b-AP15, VLX1570 and PR-619, respectively (Figure 1C). In contrast, UPS inhibition by Bortezomib and MG132 or the inhibition of the E1 ubiquitin-activating enzyme by Pyr-41 did not affect actin reorganization (Figure 1B). Our screening strategy thus identifies multiple DUB inhibitors (DUBi) as novel inducers of actin assembly.

### DUB Inhibitors Promote Actin Polymerization and Impair Leukemic Cell Migration

**FIGURE 2:**
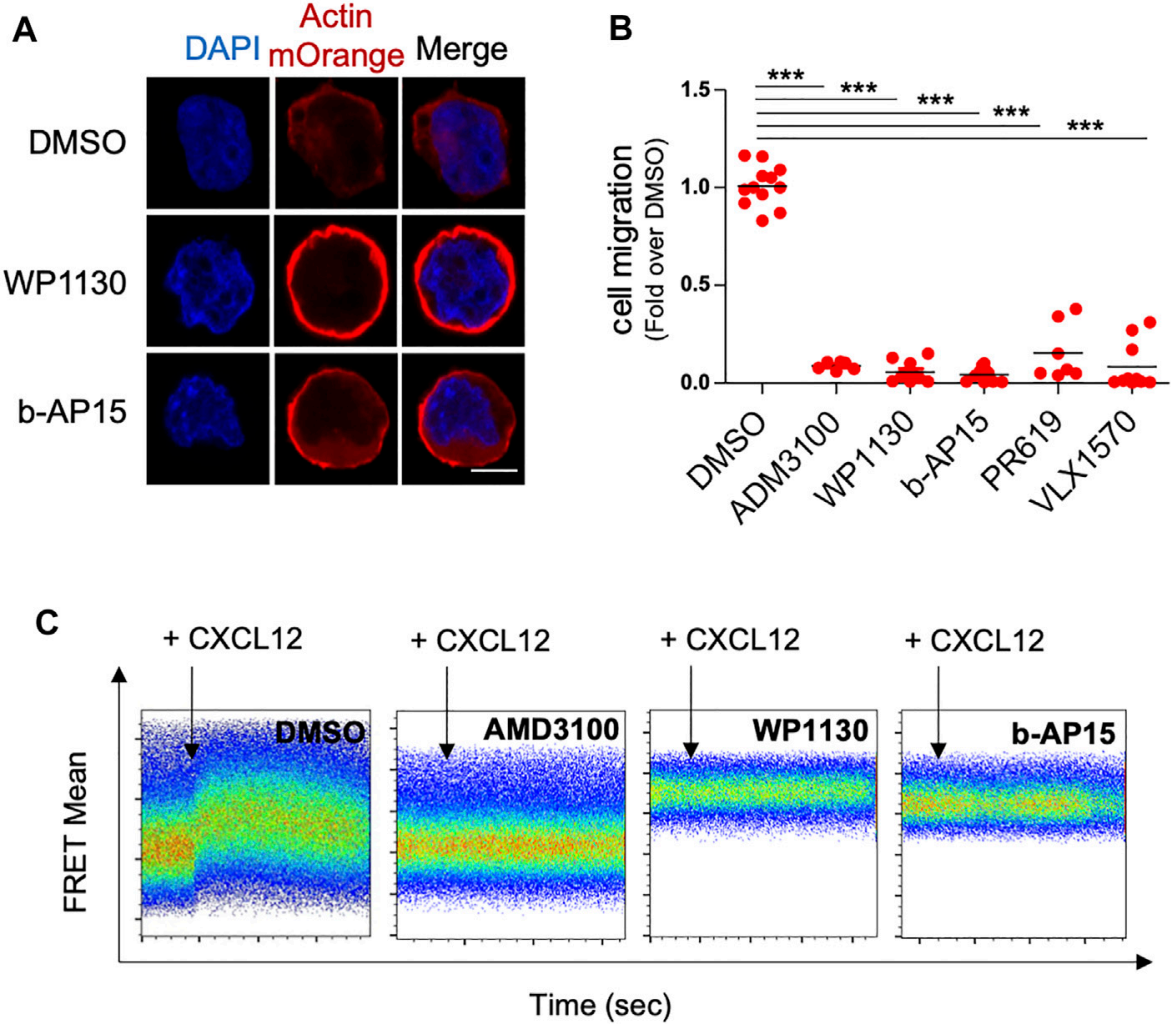
DUB inhibition increases actin polymerization and blocks leukemic cell chemotactic migration. **(A)** Confocal microscopy analysis of actin-mOrange distribution in ACT Jurkat cells treated with DMSO or 15 µM of WP1130 and b-AP15 for 30 min. Nuclei were counterstained with DAPI before analysis. Scale bar, 5 µm. **(B)** Effects of WP1130, b-AP15, PR-619 and VLX1570 on CXCL12-induced Jurkat T cell migration. Cells were migrated against 100 ng/ml of CXCL12 for 90 min in the presence or not of 15 µM of the indicated compound. Results are expressed as the fold of cell migration compared to DMSO control condition. Scatter plots show the mean of *n* = 3 independent experiments. ***p < 0.001, unpaired *t*-tests between DMSO and each treated condition. **(C)** Real time analysis of actin FRET signals induced by 100 ng/ml CXCL12 on ACT cell line treated with DMSO or 15 µM WP1130 and b-AP15 for 30 min. Flow cytometry histograms show representative data on ACT cell line treated with DMSO or DUBs inhibitors. Data are representative of *n* = 3 independent experiments. The effect of CXCR4 inhibitor AMD3100 (5 µM) on CXCL12-induced actin FRET signals is shown as control.

To confirm the effect of DUBi on actin polymerization, we analyzed ACT cells by confocal microscopy following treatment with WP1130 and b-AP15. DUB inhibition by WP1130 and b-AP15 resulted in a dramatic redistribution of actin-mOrange fluorescence in a cortical ring (Figure 2A). Actin remodeling is a crucial event regulating cell motility (Olson and Sahai, 2009; Pollard and Cooper, 2009). We thus examined the effect of DUB inhibition on leukemic T cell chemotactic migration. Jurkat cells were treated or not with DUBi and the CXCR4 inhibitor AMD3100 for 60 min and equal numbers of cells were allowed to migrate for 90 min towards a gradient of CXCL12 in Transwell chambers as described before (Ticchioni et al., 2002). The number of migrating cells in lower chambers was then determined by flow cytometry. The chemotaxis-driven migration of leukemic cells treated with WP1130, b-AP15, VLX1570, PR-619 and AMD3100 was dramatically impaired compared to cells treated with DMSO (more than 90% of reduction) (Figure 2B). Note that the dose of WP1130 and b-AP15 that blocked cell migration had no impact on Jurkat cell viability as shown by FACS analysis of annexin V/PI staining (Supplementary Figure S2). We next examined whether the inhibition of leukemic cell migration by DUBi originated from an impaired response to CXCL12 of its receptor CXCR4. Real time analysis of actin FRET following CXCL12 stimulation showed that the rapid reorganization of actin that was triggered by CXCL12 was completely abolished by a co-treatment with WP1130, b-AP15 and AMD3100 (Figure 2C). Importantly, DUB inhibition had no effect on the levels of CXCL12 receptor CXCR4 (Supplementary Figure S3). These observations show that DUB inhibition promotes exacerbated actin polymerization, which impairs leukemic cell chemotactic migration.

### DUB Inhibition Induces Actin Reorganization Through Cofilin Dephosphorylation

**FIGURE 3:**
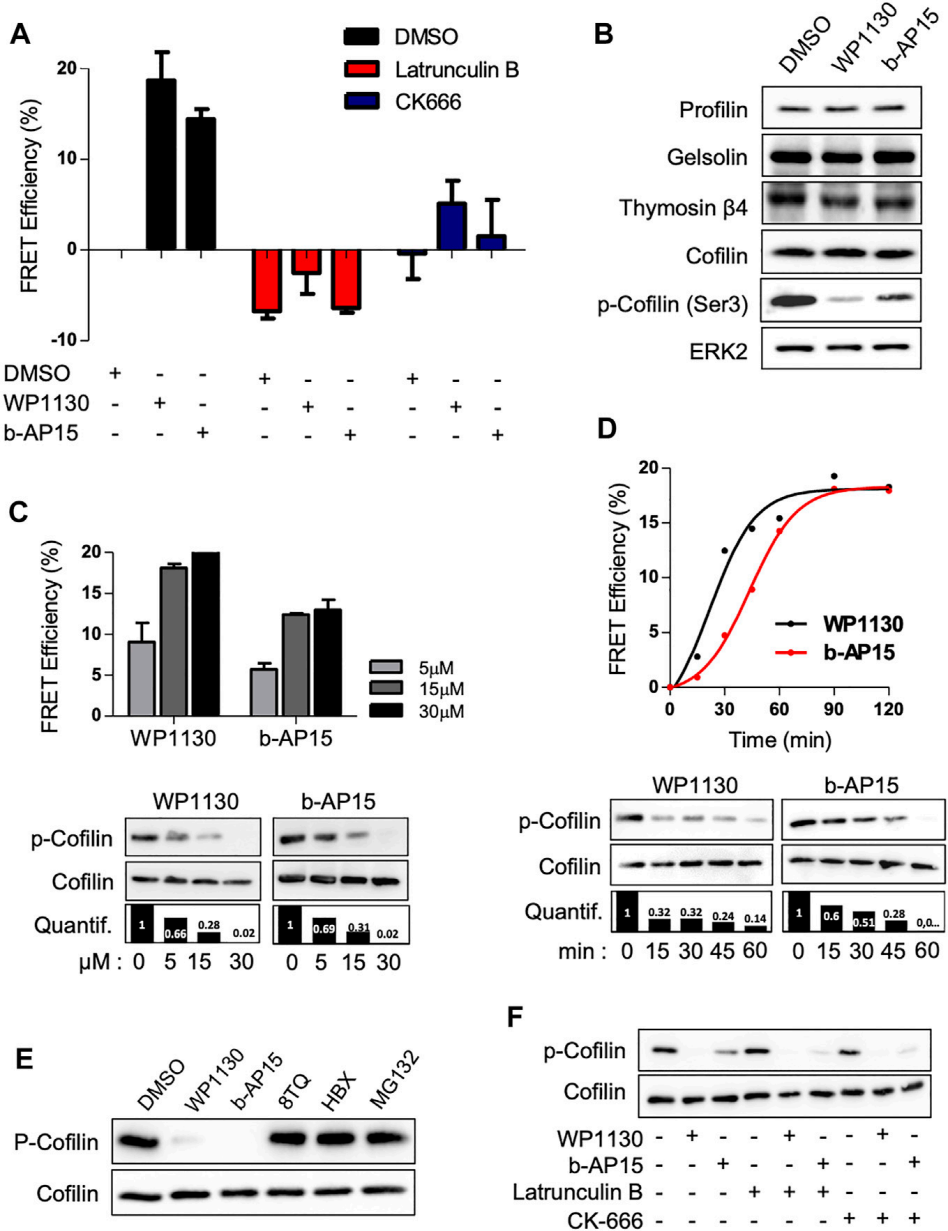
DUB inhibition induces cofilin dephosphorylation. **(A)** Analysis of actin FRET efficiencies on ACT cell line pre-treated with 500 nM Latrunculin B or 5 µM CK-666 for 1 h prior addition of 15 µM of WP1130 or b-AP15 for one additional hour. Data are expressed as the percentage of FRET efficiency between DMSO-treated and compound-treated cells. Graphs are mean ± SD of *n* = 3 independent experiments. **(B)** Immunoblot analysis showing the effect of WP1130 and b-AP15 (1 h, 15 µM) on the levels of actin sequestering proteins. Membranes were probed with the indicated antibodies and anti-ERK2 as loading control. **(C)** Effect of increasing amounts of WP1130 and b-AP15 on the phosphorylation of cofilin at Ser3. Membranes were probed with the indicated antibodies and quantified against total cofilin signal (bar graph). Upper panel, bar graphs show actin FRET efficiencies recorded on ACT cells upon the same treatment (mean ± SD of 3 independent experiments). immunoblot analysis of lysates obtained from cells treated as above. **(D)** Time-course analysis of cofilin Ser3 phosphorylation following treatment with 15 µM of WP1130 and b-AP15 for the indicated time. Membranes were probed with the indicated antibodies and quantified against total cofilin signal (bar graph). Upper panel, the graph shows the corresponding effects on actin FRET efficiencies. **(E)** WP1130 and b-AP15, but not 8TQ, HBX41108 and MG132, reduce cofilin Ser3 phosphorylation levels. Immunoblotting were performed on lysates from Jurkat cells treated with 15 µM of the indicated compound for 30 min. **(F)** Immunoblot analysis of cofilin Ser3 phosphorylation on cells treated with 500 nM Latrunculin B or 5 µM CK-666 prior addition of 15 µM of WP1130 and b-AP15 for 30 min.

We next wish to characterize how DUB inhibition resulted in actin reorganization. The Jurkat ACT cells were pre-treated with the F-actin depolymerizing drug Latrunculin B (LB) or the Arp2/3 complex inhibitor CK-666. As shown in Figure 3A, the pre-treatment with LB abolished the elevation of actin FRET signals induced by WP1130 and b-AP15, whereas the pre-treatment with CK-666 dramatically reduced it, indicating the involvement of actin monomers nucleation and/or assembly in this process. We therefore hypothesized that DUB inhibition could affect the expression and/or the activity of actin-binding protein involved in actin nucleation or polymerization (Pollard, 2016). Western blot analysis of lysates from Jurkat cells treated with WP1130 and b-AP15 revealed that DUB inhibition did not affect the protein levels of ABPs such as profilin, gelsolin, cofilin and thymosin β4. However, a decrease of the phosphorylation of cofilin serine 3 was observed upon WP1130, b-AP15, VLX1570 and PR-619 treatment (Figure 3B) (Supplementary Figure S4). The phosphorylation of the actin-severing protein cofilin suppresses its depolymerizing and nucleation activity, thereby pointing at cofilin as a key target of DUBs in actin remodeling. Dose-response and kinetic analysis of DUBi treatment further showed a correlation between cofilin dephosphorylation and actin FRET Efficiency (Figures 3C,D). In contrast, MG132 and two other DUBi, 8TQ and HBX41108, which generated no actin FRET signals (Figure 1B), had no impact on the levels of cofilin phosphorylation (Figure 3E). Importantly, Jurkat cell treatment with Latrunculin B and CK-666 did not affect DUBi-induced cofilin dephosphorylation (Figure 3F), indicating that cofilin dephosphorylation was not secondary to actin depolymerization.

**FIGURE 4:**
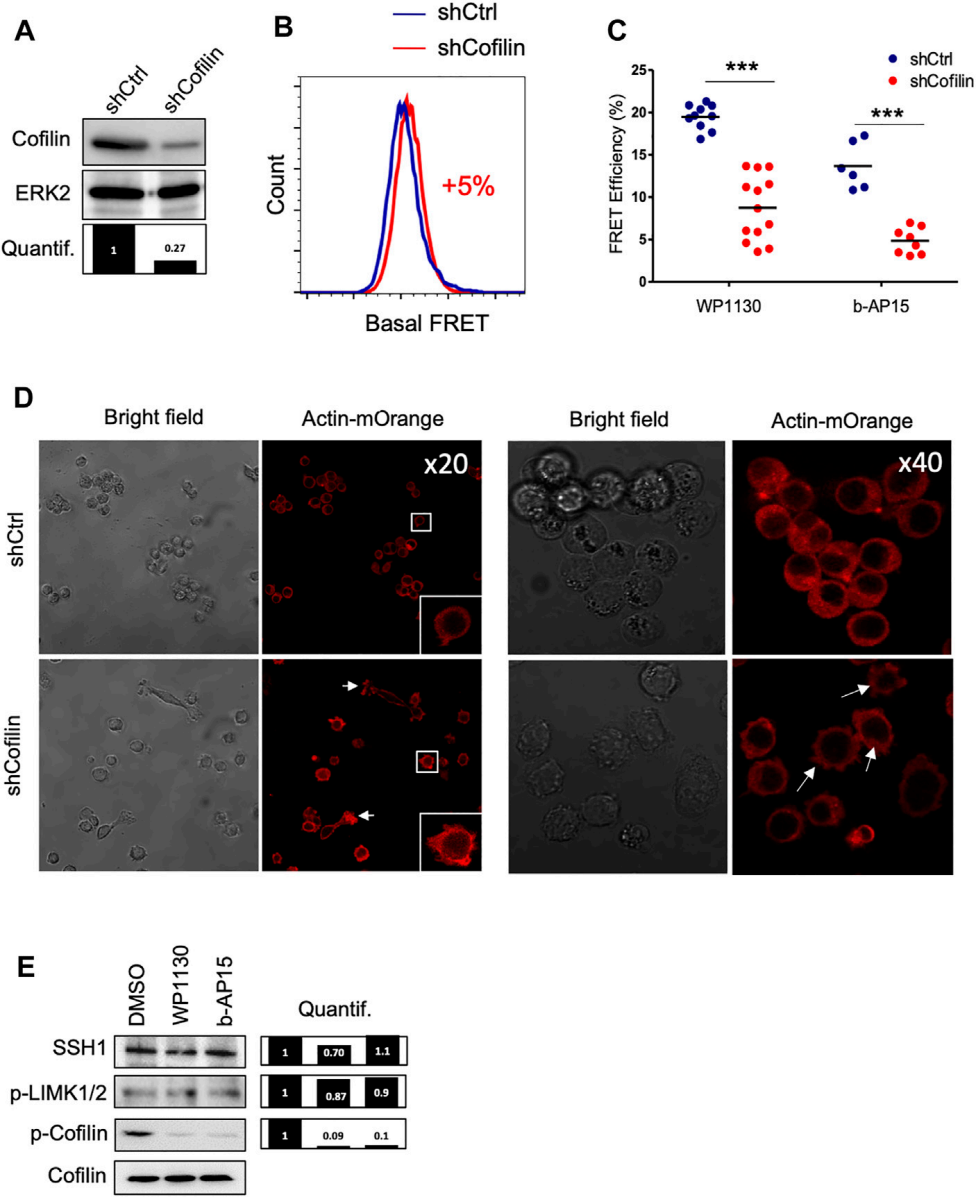
Effect of cofilin knockdown on DUBi-induced actin reorganization. **(A)** Immunoblot analysis showing cofilin downregulation on Jurkat ACT cell line following stable transduction with shCtrl or shCofilin vectors. ERK2, loading control. Densitometric quantification to ERK2 signal is shown (lower panel). **(B)** Basal actin FRET measurement expressing shCtrl or shCofilin vectors. Data are representative of *n* = 5 independent experiments. **(C)** Analysis of actin FRET efficiency following DUB inhibition on shCtrl and shCofilin ACT cell lines. Cells were treated or not with WP1130 and b-AP15 (15 µM each) for 30 min and FRET efficiencies were recorded by flow cytometry. Data are expressed as the percentage of FRET efficiency between DMSO-treated and compound-treated cells. Scatter plots show the mean of *n* = 5 independent experiments. ***p < 0.001, unpaired *t*-tests between DMSO and each treated condition. **(D)** Confocal microscopy analysis of actin-mOrange reorganization in shCtrl and shCofilin ACT cells. Scale bar, 20 µm. **(E)** Effect of WP1130 and b-AP15 on SSH1 expression and LIMK1/2 and Cofilin phosphorylation. Cells were treated for 1 h at 37°C with the indicated compound (15 µM) and lysates were analyzed by immunoblotting with the indicated antibodies and quantified by densitometry analysis against total cofilin signal (right panel).

To confirm that cofilin is a major target for cytoskeletal changes induced following DUB inhibition, we stably knock-downed cofilin in the ACT cell line (Figure 4A). Surprisingly, cofilin-depleted leukemic cells exhibited a very low modification of basal actin FRET signal (+5%) compared to control cells (Figure 4B). However, the reduction of actin FRET efficiencies observed in shCofilin ACT versus shCtrl ACT reporter cells treated with WP1130 and b-AP15 revealed that actin polymerization induced by DUBi required the expression of cofilin (from 19% for shCtrl to 9% for shCofilin treated with WP1130 and from 13 to 4% for cells treated with b-AP15) (Figure 4C). Confocal microscopy analyses further showed that cofilin depletion in Jurkat cells resulted in increased polymerization of actin filaments associated with uropod-like structures and elongated cells (Figure 4D), a phenotype that is consistent with the described role of cofilin (Nishita et al., 2005). We next examined the involvement of Slingshot Protein Phosphatase (SSH) and LIM domain kinase (LIMK), two known upstream regulators of cofilin phosphorylation and activity (Huang et al., 2006; Prunier et al., 2017). DUB inhibition with WP1130 and b-AP15 has no detectable impact on LIMK phosphorylation and SSH1 expression as measured by immunoblot analysis (Figure 4E). These results indicate that actin reorganization induced by DUBi involves a non-degradative and LIMK-independent modulation of cofilin activity.

### Actin Reorganization Induced by DUBi Is ROS-Dependent and Involves Cofilin Oxidation

**FIGURE 5:**
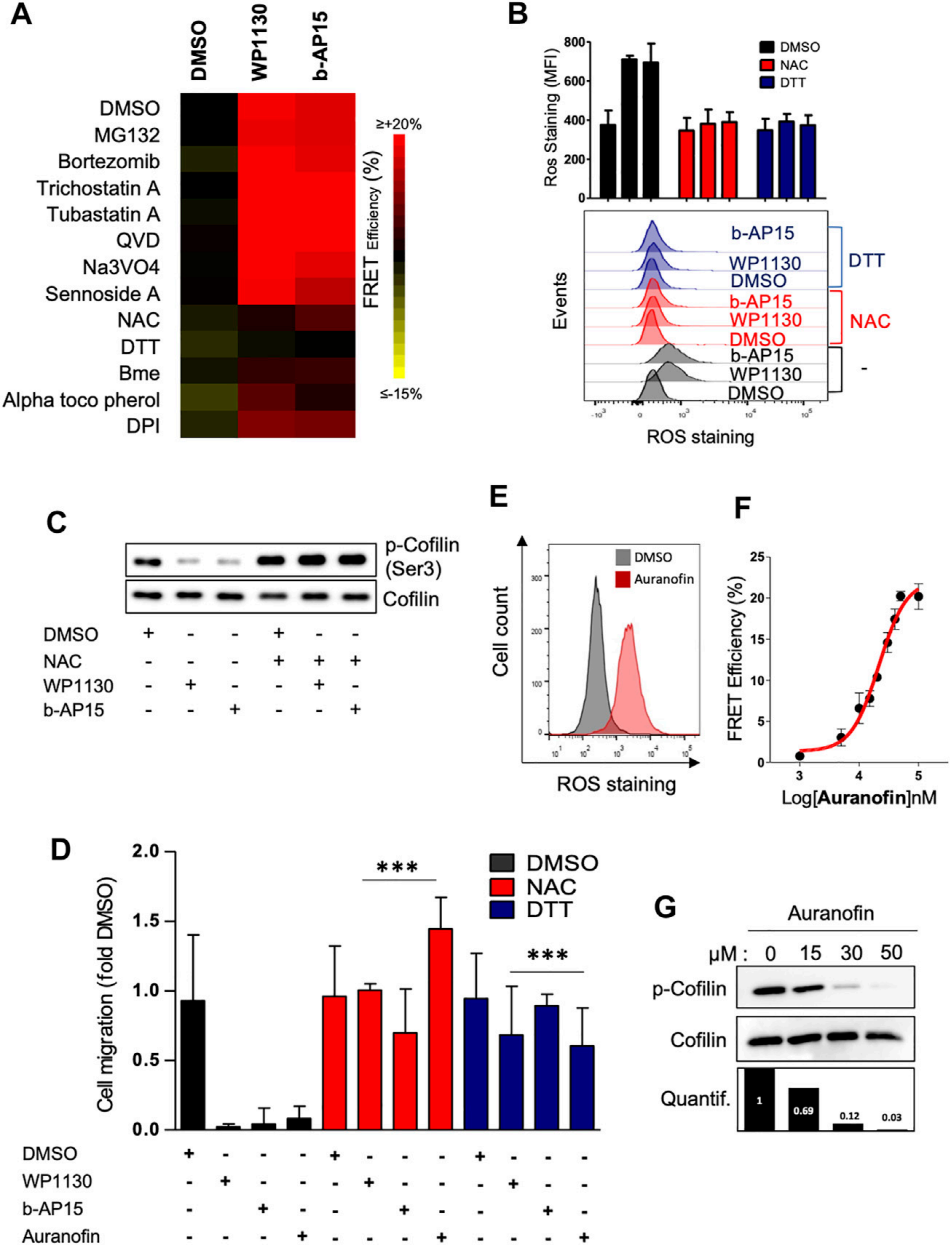
Actin cytoskeleton reorganization induced by WP1130 and b-AP15 is ROS dependent. **(A)** Heatmap representation of actin FRET efficiencies on ACT reporter cells that have been pre-treated with the indicated compounds, following exposure to 15 µM WP1130 or b-AP15 for 60 min. FRET signals were recorded by flow cytometry. Data are expressed as the percentage of FRET efficiency between DMSO-treated and compound-treated cells and are representative of *n* = 3 independent experiments. **(B)** Effect of WP1130 and b-AP15 on ROS production. ACT reporter cells that have been pre-treated with 1 mM DTT and 100 µM NAC were incubated with 15 µM WP1130 or b-AP15 for 60 min. Following treatment, cells were stained with 5 µM CM-H_2_DCFDA and analyzed by flow cytometry. Bar graphs show the mean fluorescence intensity (MFI) of ROS staining of *n* = 3 independent experiments. **(C)** Whole cell lysates of Jurkat pre-treated with 100 µM NAC before addition or not of 15 µM WP1130 or b-AP15 for 30 min were analyzed by immunoblotting with antibodies against phosphorylated cofilin and total cofilin. **(D)** Effect of WP1130, b-AP15 and Auranofin on chemotactic migration of leukemic cells pretreated or not with DTT and NAC. Cells were migrated against 100 ng/ml of CXCL12 for 90 min in the presence or not of the indicated compound. Results are expressed as the fold of cell migration compared to the DMSO condition. Bar graphs show the mean of *n* = 3 independent experiments. ***p < 0.001, unpaired *t*-tests between DMSO and each treated condition. **(E)** Effect of Auranofin on ROS production. **(F)** Dose response curve of the activity of Auranofin on actin FRET efficiency. **(G)** Immunoblot analysis showing the effect of Auranofin on the phosphorylation of cofilin at Ser3. Densitometric quantification against total cofilin signal is shown (lower panel).

We next wished to clarify the molecular mechanism underlying the effects of DUB inhibition on actin cytoskeleton. DUB inhibition is linked to increased protein ubiquitination followed by proteasome-mediated proteolytic degradation and/or aggresome formation.(Kapuria et al., 2010; D’Arcy et al., 2011). Consistently, we observed that the treatment of Jurkat cells with WP1130 and b-AP15 induced the accumulation of polyubiquitinated proteins (Supplementary Figure S5A). However, the direct inhibition of 26S proteasome by MG-132 and bortezomid had no effect on either basal (Figure 1B) or DUBi-induced actin FRET signals (Figure 5A), indicating that DUB-mediated actin regulation is not a direct consequence of the accumulation of polyubiquitinated proteins. Furthermore, in contrast to WP1130 or MG-132, b-AP15 treatment did not result in aggresome formation (Supplementary Figure S5B), suggesting that the effect of DUB inhibition on actin remodeling did not follow protein accumulation within aggresome-like structures. In support to this, blocking the regulator of aggresome formation HDAC6 (Kawaguchi et al., 2003) with tubastatin A or trichostatin A had no impact on actin assembly that was promoted by WP1130 and b-AP15 (Figure 5A) (Supplementary Figure S5C). Recent studies have linked DUB inhibition to increased oxidative stress and ROS generation (Didier et al., 2018; Charbonneau et al., 2019; Zhang et al., 2019). Conversely, DUBs can be inactivated by ROS (Cotto-Rios et al., 2012; Lee et al., 2013). Keeping in mind that cofilin activity can be modulated by oxidative signals (Klemke et al., 2008; Cameron et al., 2015), we examined the involvement of ROS in DUBi-induced actin rearrangement. The pre-treatment of Jurkat ACT cells with the antioxidant compounds DTT, NAC, α-tocopherol and DPI abrogated actin FRET signals that were induced by WP1130 and b-AP15 (Figure 5A). Consistently, the addition of DTT or NAC prevented the generation of ROS induced by DUB inhibition (Figure 5B) and the dephosphorylation of cofilin that was induced by WP1130 and b-AP15 (Figure 5C). In addition, the pretreatment of Jurkat cells with NAC or DTT restored the ability of leukemic cells to migrate against CXCL12 when DUB activity was inhibited (Figure 5D). As a control of the effect of ROS on actin reorganization in leukemic cells, the treatment with Auranofin, a compound known for its capacity to induce ROS in leukemic cells (Fiskus et al., 2014) (Figure 5E), was found to increase actin FRET signals (Figure 5F), decreased cofilin serine 3 phosphorylation (Figure 5G), and blocked CXCL12-induced chemotactic cell migration in ROS-dependent manner (Figure 5D).

**FIGURE 6:**
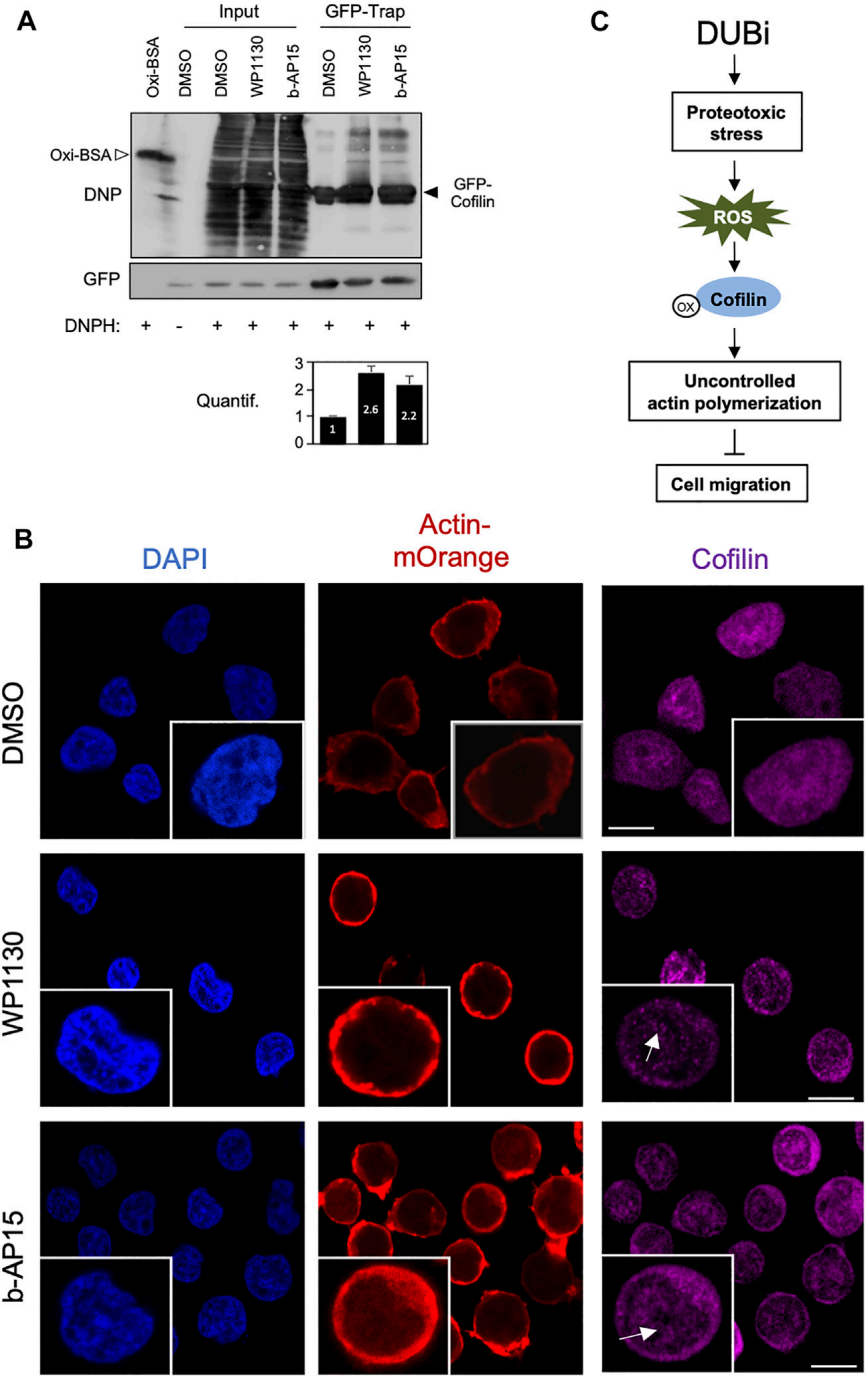
Inhibition of DUBs in leukemic cells induces Cofilin oxidation and oligomerization. **(A)** Jurkat cells stably expressing GFP-cofilin were treated or not with 15 µM WP1130 or b-AP15 for 30 min and lysed. Cell lysates were incubated with anti-GFP nanobody (GFP-Trap) coupled to agarose beads. The trapped proteins and a fraction of the cell lysates (Input) were incubated with DNPH for 15 min at room temperature before immunoblot with antibodies against DNP or GFP. Anti-DNP immunoblot of DNP-labelled oxidized BSA (40 ng) is shown as a control. Data are representative of *n* = 2 experiments. Densitometric quantification of cofilin oxidation against GFP signal is shown as bar graph (bottom panel). **(B)** Immunofluorescence microscopy analysis of actin-mOrange and Cofilin distribution in ACT Jurkat cell treated with DMSO or 15 µM of WP1130 and b-AP15 for 30 min. Scale bar, 10 µm. Inset, white arrows show Cofilin aggregates. **(C)** Schematic overview of the effect of DUB inhibition on cofilin activity, actin polymerization and cell migration.

These results showing that DUBs regulate cofilin function through a ROS-dependent pathway led us to investigate the impact of DUB inhibition on cofilin oxidation, a process linked to actin cytoskeleton dynamics in T cells (Samstag et al., 2013). Jurkat cells stably expressing a GFP-cofilin construct were treated with WP1130 or b-AP15 for 30 min. Oxidated proteins were labeled with DNPH in cell lysates after pulldown with anti-GFP-Trap nanobodies and immunoblot analysis with antibodies against DNP. As shown in Figure 6A, WP1130 and b-AP15 increased cofilin oxidation by 2.6 and 2.2 fold, respectively, compared to DMSO-treated conditions. Cofilin exhibits a tendency for self-association under oxidative stress, inducing the rapid formation of dimers and oligomers (Pfannstiel et al., 2001; Goyal et al., 2013). Consistently, immunofluorescence microscopy analysis revealed that compared to control cells Jurkat cells treated with DUBi exhibit increased cofilin aggregates that partially located in the nucleus (Figure 6B). Together, these results demonstrate that DUBs inhibitors display antimigratory activity in leukemic cells through ROS-mediated cofilin oxidation and actin cytoskeleton dysfunction.

## Discussion

The remodelling of the actin cytoskeleton is modulated by interactions between actin and a number of proteins such as ABPs (Pollard, 2016). The expression, activity and location of actin and ABPs are regulated by a large number of PTMs (Terman and Kashina, 2013), which largely account for the complexity of actin networks and their dysregulation in pathological processes. A better understanding of these molecular mechanisms is therefore of critical importance. In this study, we have examined the impact of pharmacological modulators of PTMs on actin dynamics by using our method named Actin CytoFRET (Larbret et al., 2013). The screening with Actin CytoFRET of a small library of compounds targeting enzymes involved in acetylation, methylation, phosphorylation and ubiquitination of proteins revealed that several small molecules, including WP1130, b-AP15, VLX1570 and PR-619, have a marked effect on actin polymerization. These compounds have been characterized as broad-range inhibitors of DUBs, a group of enzymes with a cysteine protease activity, which catalyze the removal of ubiquitin or ubiquitin chains from post-translationally modified targets (Kapuria et al., 2010; Altun et al., 2011; D’Arcy et al., 2011). DUBi can form stable covalent adducts with specific cysteine contained within the catalytic domain of these enzymes, thereby inhibiting their cysteine protease activity. DUBs display several essential cellular functions, such as the recycling of ubiquitin, protein trafficking, and regulating protein degradation by the ubiquitin-proteasome system (UPS) (Komander et al., 2009; D’Arcy et al., 2015). To this regard, the effects of DUB inhibitors on actin remodeling is unlikely related to a general inhibition of the UPS since short term proteasomal inhibition by Bortezomib and MG132 does not alter actin reorganization. In addition, other DUB inhibitors such as 8TQ (Mofers et al., 2017) and HBX41108 (Colombo et al., 2010), as well as the selective inhibitor of ubiquitin-activating enzyme E1 PYR-41 (Yang et al., 2007), have no detectable impact on actin dynamics, indicating that the identified DUB inhibitors are not affecting actin polymerization through a general disruption of ubiquitin homeostasis or proteasome activity.

Molecular mechanisms that are considered hallmarks of DUB inhibition consist of an accumulation of poly-ubiquitinated proteins, the activation of the unfolded protein response (UPR), proteotoxic and oxidative stress, and cytotoxicity (D'Arcy et al., 2011; Brnjic et al., 2014; Tian et al., 2014; Didier et al., 2018; Zhang et al., 2019). Whether DUB inhibitors can regulate others cellular mechanisms has been poorly investigated. We found that short exposure of leukemic cells to DUB inhibitors increased actin remodeling and dramatically impedes leukemic cell migration through a ROS-dependent process. Such a mechanism was supported by the observation that anti-oxidant molecules prevented the exacerbated actin remodeling triggered following DUB inhibition. Although the induction of ROS is a common feature in response to these compounds, what the generated ROS exactly target is poorly characterized. Our results show that enhanced oxidation of cofilin represents another major determinant of the cellular action of DUBi in leukemic cells. The treatment of Jurkat cells with DUB inhibitors such as WP1130, b-AP15, VLX1570 and PR-619 was associated with a marked downregulation of phosphorylated levels of cofilin serine 3. In addition, the induction of actin FRET signals by DUBi is markedly reduced in Jurkat biosensor cells in which cofilin has been knocked down. The ADF/cofilin family are closely related ABPs that play overlapping but distinct roles in cellular processes in which rapid reorganization or renewal of actin filaments is required (Bernstein and Bamburg, 2010; Kanellos and Frame, 2016). The activity of ADF/cofilin family is tightly regulated by phosphorylation of the common serine 3 residue by upstream kinases such as LIMK. Unphosphorylated cofilin bind actin filaments and promote cycles of actin depolymerization/polymerization that are critical for biological processes (Kanellos and Frame, 2016). Interestingly, the treatment with WP1130 and b-AP15, in addition to induce the dephosphorylation of cofilin on serine 3, also promotes cofilin oxidation. We also show that LIMK and SSH1 were not affected following DUB inhibition, indicating that cofilin dephosphorylation occurs independently of SSH/LIMK modulation. Consistent with our observations, cofilin oxidation in T cells has been associated with an increase of F-actin characterized by cell migration defects and hyporesponsiveness (Klemke et al., 2008), leading to the hypothesis that cofilin acts as a redox sensitive mediator of actin dynamics in T cell (Samstag et al., 2013). Furthermore, polarized migration of mesenchymal cells has been linked with localized H2O2 production that inhibits cofilin via cysteine oxidation (Cameron et al., 2015; Rudzka et al., 2015). Our hypothesis is that, while cofilin oxidation following DUB inhibition promotes its dephosphorylation and binding to F-Actin, it also prevents its severing activity, leading to an uncontrolled production of actin filaments, a massive accumulation of cortical actin, which eventually impairs leukemic cell migration (Figure 6C).

The notion that ROS produced in response to DUB inhibitors are central to their action on actin cytoskeleton is consistent with our observations that the treatment with the thioredoxin reductase inhibitor Auranofin, which induces a strong oxidative stress, is also accompanied by actin remodeling and impaired leukemic cell migration. The oxidation of the catalytic cysteine of DUBs by exogenous and endogenous ROS have been shown to reversibly inhibit their isopeptidase activity (Lee et al., 2013). It is therefore possible that DUB inhibitors may have their action amplified through oxidation of cysteine residues within the catalytic domain of DUBs, therefore reinforcing their potent action on cell migration. In this context, it would be interesting to determine how the expression of oxidation-resistant cofilin may affect actin dynamics and leukemic cell migration.

The critical role of ROS as second messengers in cell biological functions including actin cytoskeleton remodeling and cell migration has been described (Finkel, 2011; Stanley et al., 2014). Here, we identify several inhibitors of deubiquitinating enzymes as critical regulators of actin cytoskeleton and cell migration through ROS-mediated regulation of the actin remodeling protein cofilin, suggesting that pharmacological targeting of DUBs may represent a novel way to manipulate leukemic cell migration.

## Data Availability

The original contributions presented in the study are included in the article/Supplementary Material, further inquiries can be directed to the corresponding authors.

## References

[B1] Altun M. Kramer H. B. Willems L. I. McDermott J. L. Leach C. A. Goldenberg S. J. (2011). Activity-based Chemical Proteomics Accelerates Inhibitor Development for Deubiquitylating Enzymes. Chem. Biol. 18 (11), 1401–1412. 10.1016/j.chembiol.2011.08.018 22118674

[B2] Badour K. Zhang J. Shi F. Leng Y. Collins M. Siminovitch K. A. (2004). Fyn and PTP-PEST-Mediated Regulation of Wiskott-Aldrich Syndrome Protein (WASp) Tyrosine Phosphorylation Is Required for Coupling T Cell Antigen Receptor Engagement to WASp Effector Function and T Cell Activation. J. Exp. Med. 199 (1), 99–112. 10.1084/jem.20030976 14707117 PMC1887720

[B3] Bernstein B. W. Bamburg J. R. (2010). ADF/cofilin: a Functional Node in Cell Biology. Trends Cel Biol 20 (4), 187–195. 10.1016/j.tcb.2010.01.001 PMC2849908 20133134

[B4] Brnjic S. Mazurkiewicz M. Fryknäs M. Sun C. Zhang X. Larsson R. (2014). Induction of Tumor Cell Apoptosis by a Proteasome Deubiquitinase Inhibitor Is Associated with Oxidative Stress. Antioxid. Redox Signal. 21 (17), 2271–2285. 10.1089/ars.2013.5322 24011031 PMC4241954

[B5] Cameron J. M. Gabrielsen M. Chim Y. H. Munro J. McGhee E. J. Sumpton D. (2015). Polarized Cell Motility Induces Hydrogen Peroxide to Inhibit Cofilin via Cysteine Oxidation. Curr. Biol. 25 (11), 1520–1525. 10.1016/j.cub.2015.04.020 25981793 PMC4454775

[B6] Charbonneau M. E. Passalacqua K. D. Hagen S. E. Showalter H. D. Wobus C. E. O'Riordan M. X. D. (2019). Perturbation of Ubiquitin Homeostasis Promotes Macrophage Oxidative Defenses. Sci. Rep. 9 (1), 10245. 10.1038/s41598-019-46526-9 31308397 PMC6629656

[B7] Colombo M. Vallese S. Peretto I. Jacq X. Rain J. C. Colland F. (2010). Synthesis and Biological Evaluation of 9-Oxo-9h-Indeno[1,2-B]pyrazine-2,3-Dicarbonitrile Analogues as Potential Inhibitors of Deubiquitinating Enzymes. ChemMedChem 5 (4), 552–558. 10.1002/cmdc.200900409 20186914

[B8] Cory G. O. Cramer R. Blanchoin L. Ridley A. J. (2003). Phosphorylation of the WASP-VCA Domain Increases its Affinity for the Arp2/3 Complex and Enhances Actin Polymerization by WASP. Mol. Cel 11 (5), 1229–1239. 10.1016/s1097-2765(03)00172-2 12769847

[B9] Cotto-Rios X. M. Békés M. Chapman J. Ueberheide B. Huang T. T. (2012). Deubiquitinases as a Signaling Target of Oxidative Stress. Cell Rep 2 (6), 1475–1484. 10.1016/j.celrep.2012.11.011 23219552 PMC3534866

[B10] D'Arcy P. Brnjic S. Olofsson M. H. Fryknäs M. Lindsten K. De Cesare M. (2011). Inhibition of Proteasome Deubiquitinating Activity as a New Cancer Therapy. Nat. Med. 17 (12), 1636–1640. 10.1038/nm.2536 22057347

[B11] D'Arcy P. Wang X. Linder S. (2015). Deubiquitinase Inhibition as a Cancer Therapeutic Strategy. Pharmacol. Ther. 147, 32–54. 10.1016/j.pharmthera.2014.11.002 25444757

[B12] Didier R. Mallavialle A. Ben Jouira R. Domdom M. A. Tichet M. Auberger P. (2018). Targeting the Proteasome-Associated Deubiquitinating Enzyme USP14 Impairs Melanoma Cell Survival and Overcomes Resistance to MAPK-Targeting Therapies. Mol. Cancer Ther. 17 (7), 1416–1429. 10.1158/1535-7163.mct-17-0919 29703842

[B13] Finkel T. (2011). Signal Transduction by Reactive Oxygen Species. J. Cel Biol 194 (1), 7–15. 10.1083/jcb.201102095 PMC3135394 21746850

[B14] Fiskus W. Saba N. Shen M. Ghias M. Liu J. Gupta S. D. (2014). Auranofin Induces Lethal Oxidative and Endoplasmic Reticulum Stress and Exerts Potent Preclinical Activity against Chronic Lymphocytic Leukemia. Cancer Res. 74 (9), 2520–2532. 10.1158/0008-5472.CAN-13-2033 24599128 PMC4172421

[B15] Goyal P. Pandey D. Brünnert D. Hammer E. Zygmunt M. Siess W. (2013). Cofilin Oligomer Formation Occurs *In Vivo* and Is Regulated by Cofilin Phosphorylation. PLoS One 8 (8), e71769. 10.1371/journal.pone.0071769 23951242 PMC3738525

[B16] Hao Y. H. Doyle J. M. Ramanathan S. Gomez T. S. Jia D. Xu M. (2013). Regulation of WASH-dependent Actin Polymerization and Protein Trafficking by Ubiquitination. Cell 152 (5), 1051–1064. 10.1016/j.cell.2013.01.051 23452853 PMC3640276

[B17] Huang T. Y. DerMardirossian C. Bokoch G. M. (2006). Cofilin Phosphatases and Regulation of Actin Dynamics. Curr. Opin. Cel Biol 18 (1), 26–31. 10.1016/j.ceb.2005.11.005 16337782

[B18] Kanellos G. Frame M. C. (2016). Cellular Functions of the ADF/cofilin Family at a Glance. J. Cel Sci 129 (17), 3211–3218. 10.1242/jcs.187849 27505888

[B19] Kapuria V. Peterson L. F. Fang D. Bornmann W. G. Talpaz M. Donato N. J. (2010). Deubiquitinase Inhibition by Small-Molecule WP1130 Triggers Aggresome Formation and Tumor Cell Apoptosis. Cancer Res. 70 (22), 9265–9276. 10.1158/0008-5472.CAN-10-1530 21045142

[B20] Kawaguchi Y. Kovacs J. J. McLaurin A. Vance J. M. Ito A. Yao T. P. (2003). The Deacetylase HDAC6 Regulates Aggresome Formation and Cell Viability in Response to Misfolded Protein Stress. Cell 115 (6), 727–738. 10.1016/s0092-8674(03)00939-5 14675537

[B21] Klemke M. Wabnitz G. H. Funke F. Funk B. Kirchgessner H. Samstag Y. (2008). Oxidation of Cofilin Mediates T Cell Hyporesponsiveness under Oxidative Stress Conditions. Immunity 29 (3), 404–413. 10.1016/j.immuni.2008.06.016 18771940

[B22] Komander D. Clague M. J. Urbé S. (2009). Breaking the Chains: Structure and Function of the Deubiquitinases. Nat. Rev. Mol. Cel Biol 10 (8), 550–563. 10.1038/nrm2731 19626045

[B23] Lang P. Gesbert F. Delespine-Carmagnat M. Stancou R. Pouchelet M. Bertoglio J. (1996). Protein Kinase A Phosphorylation of RhoA Mediates the Morphological and Functional Effects of Cyclic AMP in Cytotoxic Lymphocytes. EMBO J. 15 (3), 510–519. 10.1002/j.1460-2075.1996.tb00383.x 8599934 PMC449969

[B24] Larbret F. Dubois N. Brau F. Guillemot E. Mahiddine K. Tartare-Deckert S. (2013). Technical advance: Actin CytoFRET, a Novel FRET Flow Cytometry Method for Detection of Actin Dynamics in Resting and Activated T Cell. J. Leukoc. Biol. 94 (3), 531–539. 10.1189/jlb.0113022 23794712

[B25] Lee J. G. Baek K. Soetandyo N. Ye Y. (2013). Reversible Inactivation of Deubiquitinases by Reactive Oxygen Species *In Vitro* and in Cells. Nat. Commun. 4, 1568. 10.1038/ncomms2532 23463011 PMC3615374

[B26] Mofers A. Pellegrini P. Linder S. D'Arcy P. (2017). Proteasome-associated Deubiquitinases and Cancer. Cancer Metastasis Rev. 36 (4), 635–653. 10.1007/s10555-017-9697-6 29134486 PMC5721125

[B27] Nishita M. Tomizawa C. Yamamoto M. Horita Y. Ohashi K. Mizuno K. (2005). Spatial and Temporal Regulation of Cofilin Activity by LIM Kinase and Slingshot Is Critical for Directional Cell Migration. J. Cel Biol 171 (2), 349–359. 10.1083/jcb.200504029 PMC2171197 16230460

[B28] Olson M. F. Sahai E. (2009). The Actin Cytoskeleton in Cancer Cell Motility. Clin. Exp. Metastasis 26 (4), 273–287. 10.1007/s10585-008-9174-2 18498004

[B29] Paavilainen V. O. Bertling E. Falck S. Lappalainen P. (2004). Regulation of Cytoskeletal Dynamics by Actin-Monomer-Binding Proteins. Trends Cel Biol 14 (7), 386–394. 10.1016/j.tcb.2004.05.002 15246432

[B30] Pfannstiel J. Cyrklaff M. Habermann A. Stoeva S. Griffiths G. Shoeman R. (2001). Human Cofilin Forms Oligomers Exhibiting Actin Bundling Activity. J. Biol. Chem. 276 (52), 49476–49484. 10.1074/jbc.M104760200 11679578

[B31] Pollard T. D. (2016). Actin and Actin-Binding Proteins. Cold Spring Harb Perspect. Biol. 8 (8), a018226. 10.1101/cshperspect.a018226 26988969 PMC4968159

[B32] Pollard T. D. Cooper J. A. (2009). Actin, a central Player in Cell Shape and Movement. Science 326 (5957), 1208–1212. 10.1126/science.1175862 19965462 PMC3677050

[B33] Prunier C. Prudent R. Kapur R. Sadoul K. Lafanechère L. (2017). LIM Kinases: Cofilin and beyond. Oncotarget 8 (25), 41749–41763. 10.18632/oncotarget.16978 28445157 PMC5522193

[B34] Rotty J. D. Wu C. Bear J. E. (2013). New Insights into the Regulation and Cellular Functions of the ARP2/3 Complex. Nat. Rev. Mol. Cel Biol 14 (1), 7–12. 10.1038/nrm3492 23212475

[B35] Rudzka D. A. Cameron J. M. Olson M. F. (2015). Reactive Oxygen Species and Hydrogen Peroxide Generation in Cell Migration. Commun. Integr. Biol. 8 (5), e1074360. 10.1080/19420889.2015.1074360 27066166 PMC4802769

[B36] Samstag Y. John I. Wabnitz G. H. (2013). Cofilin: a Redox Sensitive Mediator of Actin Dynamics during T-Cell Activation and Migration. Immunol. Rev. 256 (1), 30–47. 10.1111/imr.12115 24117811 PMC3884758

[B37] Schaefer A. Nethe M. Hordijk P. L. (2012). Ubiquitin Links to Cytoskeletal Dynamics, Cell Adhesion and Migration. Biochem. J. 442 (1), 13–25. 10.1042/BJ20111815 22280013

[B38] Sit S. T. Manser E. (2011). Rho GTPases and Their Role in Organizing the Actin Cytoskeleton. J. Cel Sci 124 (Pt 5), 679–683. 10.1242/jcs.064964 21321325

[B39] Stanley A. Thompson K. Hynes A. Brakebusch C. Quondamatteo F. (2014). NADPH Oxidase Complex-Derived Reactive Oxygen Species, the Actin Cytoskeleton, and Rho GTPases in Cell Migration. Antioxid. Redox Signal. 20 (13), 2026–2042. 10.1089/ars.2013.5713 24251358

[B40] Suarez C. Kovar D. R. (2016). Internetwork Competition for Monomers Governs Actin Cytoskeleton Organization. Nat. Rev. Mol. Cel Biol 17 (12), 799–810. 10.1038/nrm.2016.106 PMC5125073 27625321

[B41] Terman J. R. Kashina A. (2013). Post-translational Modification and Regulation of Actin. Curr. Opin. Cel Biol 25 (1), 30–38. 10.1016/j.ceb.2012.10.009 PMC3578039 23195437

[B42] Tian Z. D'Arcy P. Wang X. Ray A. Tai Y. T. Hu Y. (2014). A Novel Small Molecule Inhibitor of Deubiquitylating Enzyme USP14 and UCHL5 Induces Apoptosis in Multiple Myeloma and Overcomes Bortezomib Resistance. Blood 123 (5), 706–716. 10.1182/blood-2013-05-500033 24319254 PMC3907756

[B43] Ticchioni M. Charvet C. Noraz N. Lamy L. Steinberg M. Bernard A. (2002). Signaling through ZAP-70 Is Required for CXCL12-Mediated T-Cell Transendothelial Migration. Blood 99 (9), 3111–3118. 10.1182/blood.v99.9.3111 11964272

[B44] Tojkander S. Gateva G. Lappalainen P. (2012). Actin Stress Fibers-Aassembly, Dynamics and Biological Roles. J. Cel Sci 125 (Pt 8), 1855–1864. 10.1242/jcs.098087 22544950

[B45] Wang H. R. Zhang Y. Ozdamar B. Ogunjimi A. A. Alexandrova E. Thomsen G. H. (2003). Regulation of Cell Polarity and Protrusion Formation by Targeting RhoA for Degradation. Science 302 (5651), 1775–1779. 10.1126/science.1090772 14657501

[B46] Yang Y. Kitagaki J. Dai R. M. Tsai Y. C. Lorick K. L. Ludwig R. L. (2007). Inhibitors of Ubiquitin-Activating Enzyme (E1), a New Class of Potential Cancer Therapeutics. Cancer Res. 67 (19), 9472–9481. 10.1158/0008-5472.CAN-07-0568 17909057

[B47] Yuan W. C. Lee Y. R. Lin S. Y. Chang L. Y. Tan Y. P. Hung C. C. (2014). K33-Linked Polyubiquitination of Coronin 7 by Cul3-KLHL20 Ubiquitin E3 Ligase Regulates Protein Trafficking. Mol. Cel 54 (4), 586–600. 10.1016/j.molcel.2014.03.035 24768539

[B48] Zhang X. Espinosa B. Saei A. A. D'Arcy P. Zubarev R. A. Linder S. (2019). Oxidative Stress Induced by the Deubiquitinase Inhibitor B-AP15 Is Associated with Mitochondrial Impairment. Oxid Med. Cel Longev 2019, 1659468. 10.1155/2019/1659468 PMC6590552 31281566

