# Supplementary material for: Deubiquitinase Inhibitors Impair Leukemic Cell Migration Through Cofilin Oxidation and Alteration of Actin Reorganization
Source: Front Pharmacol. 2022 Jan 7;12:778216. doi: 10.3389/fphar.2021.778216 (PMC8782157; doi:10.3389/fphar.2021.778216)

SUPPLEMENTARY FIGURE LEGENDS

Supplementary Figure S1. Chemical structure of DUBs inhibitors inducing actin remodelling. Chemical structure of the four compounds showing increase FRET signals, WP1130, b-AP15, VLX570 and PR619.

Supplementary Figure S2. Flow cytometry analysis of Annexin V / propidium iodide staining on Jurkat cells treated with WP1130 or b-AP15 (15µM each) for 1h Percentage of viable (AV-/PI-), early apoptotic (AV+/PI-), late apoptotic (AV+/PI-+) and necrotic (AV-/PI+) cell populations are shown in each appropriate quadrant. Percentage represents average of 3 independent experiments.

Supplementary Figure S3. DUB inhibition had no effect on the levels of CXCL12 receptor CXCR4. Flow cytometry histogram showing cell membrane CXCR4 expression level on Jurkat cells treated with DMSO or WP1130 and b-AP15.

Supplementary Figure S4. Immunoblot showing cofilin phosphorylation following treatment with VLX570 and PR619.

Supplementary Figure S5. Effect of DUB inhibition on aggresome formation. (A) Whole cell lysates of Jurkat treated with 15µM of WP1130 or b-AP15 for 60 min were analyzed by immunoblotting with antibodies against K48-linked specific polyubiquitinated chains. (B) Jurkat cells were treated with DMSO, MG132 (10µM for 6h), WP1130 (15µM for 1h) or b-AP15 (15µM for 1h) and aggresome were stained and analyzed by flow cytometry. (C) Effect of Tubastatin A (30µM) pre-treatment on Jurkat cells treated by WP1130 and b-AP15 (15µM each). Anti-acetyl tubulin immunoblot is shown as a control for Tubastatin A action.

Supplementary Figure S1

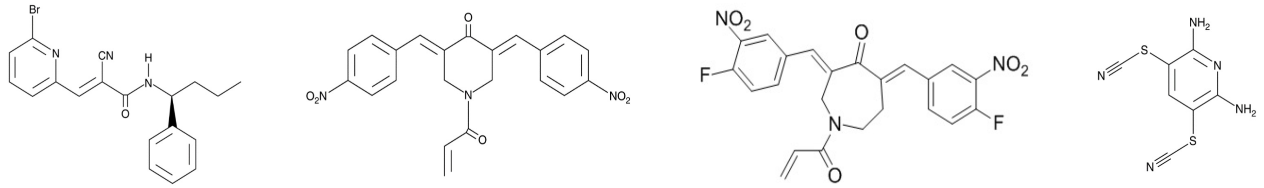

WP1130 b-AP15 VLX1570 PR-619

Supplementary Figure S2

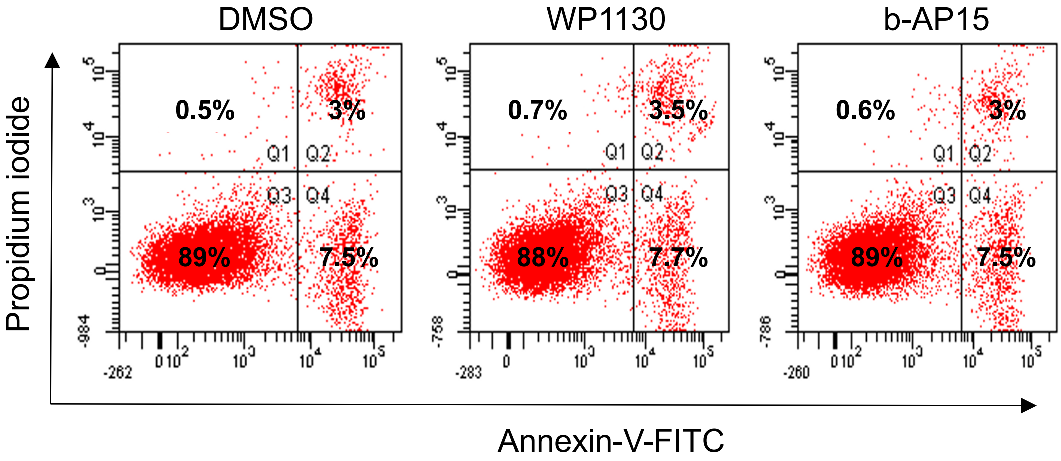

Supplementary Figure S3

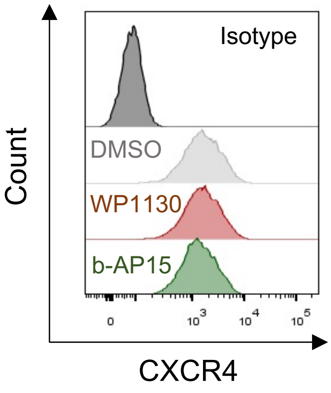

Supplementary Figure S4

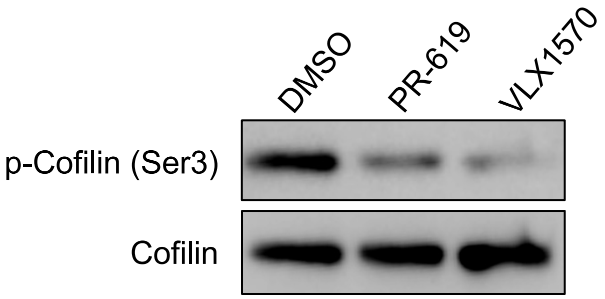

Supplementary Figure S5

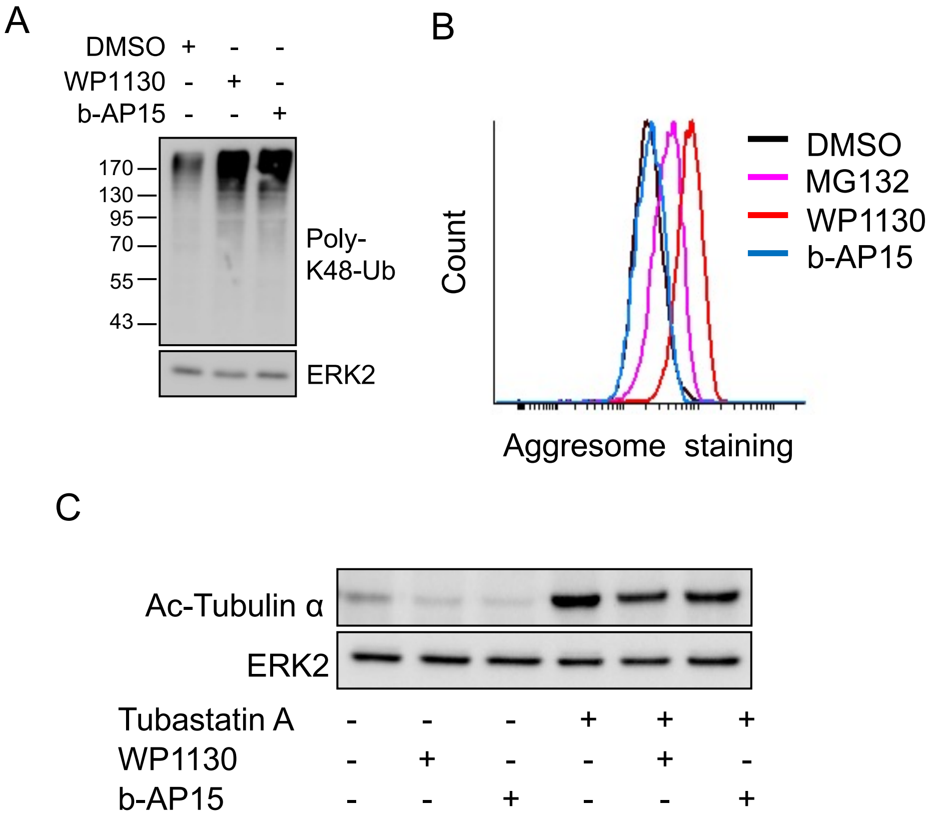

Supplement: Supplementary file 1 [file DataSheet1.PDF]
